# Achieving COVID‐19 herd immunity in Algeria: Current state of vaccination, challenges, and recommendations

**DOI:** 10.1002/puh2.76

**Published:** 2023-03-17

**Authors:** Chijioke Christopher Uhegwu, Oyinloye Emmanuel Abiodun, Usman Abubakar Haruna, Babaz Younes, Bakir Moussaouli, Goodness Ogeyi Odey

**Affiliations:** ^1^ Department of Microbiology Federal University Otuoke Otuoke Bayelsa Nigeria; ^2^ Department of Microbiology Federal University Oye‐Ekiti Oye Ekiti Nigeria; ^3^ Department of Biomedical Sciences Nazarbayev University School of Medicine (NUSOM) Astana Kazakhstan; ^4^ Department of Agronomic Sciences, Faculty of Natural and Life Sciences and Earth Sciences Kasdi Merbah University Ouargla Algeria; ^5^ Department of Agronomy, Faculty of Natural and Life Sciences University of Ghardaia Bounoura Algeria; ^6^ Department of Public Health College of Medicine University of Calabar Calabar Nigeria

**Keywords:** Algeria, COVID‐19, health policy, herd immunity, pandemic, vaccines, vaccination

## Abstract

The COVID‐19 pandemic has presented Algeria with a slew of issues, including major economic consequences. Despite implementing containment measures, the government has been unable to fully restrict the spread of the virus. To reestablish normalcy and resume economic activities, the government must discover a means to cohabit with the virus, which requires achieving herd immunity. The World Health Organization (WHO) had set a target for 70% of the population to be fully vaccinated by the end of July 2022 in order to achieve herd immunity. However, whereas vaccines are the preferred method for achieving herd immunity, Algeria has only managed to vaccinate 30.2% of its population, with only 13.5% being fully vaccinated, falling significantly short of the vaccination targets set by the WHO. This is due to vaccine hesitancy and a lack of effective vaccine distribution technology. To reach herd immunity levels, the government must gain and maintain public trust in vaccinations and improve vaccine delivery to hard‐to‐reach areas. This paper provides an overview of the current COVID‐19 situation in Algeria, progress made, and challenges toward achieving herd immunity, and recommends solutions for policymakers to develop sustainable interventions.

## INTRODUCTION

Algeria, a country located in North Africa with a population of approximately 42 million, has a public health system managed by the Ministry of Health. The Ministry is responsible for providing health care services to the population. With the emergence of COVID‐19, countries across the globe are struggling to contain the virus. One important concept to stop the spread of infections in public health is "herd immunity," which refers to indirect protection from infectious diseases resulting from a high level of immunity in the population. Herd immunity can be achieved through recovery from infection with a pathogen or through vaccination [1]. On 25 February 2020, the Ministry of Health, Population, and Hospital Reform announced the first confirmed case of COVID‐19 in Algeria, making it the second case reported in Africa, following the first case in Egypt [2].

Since then, the Algerian government implemented several measures to combat the spread of COVID‐19, including rules prohibiting gatherings of more than two people, the closure of international flights and borders, curfews, and directives confining 50% of the public workforce to their homes [3]. Despite initial beliefs that the virus would not spread in Algeria due to its sweltering climate, the health care system has been overwhelmed by an unprecedented demand for diagnostics and treatment, and health care providers are under pressure to contain the virus [2]. The pandemic has greatly affected the economy, with the suspension of flights and hotel services devastating the tourism sector [4, 5]. The cumulative impact of COVID‐19 has limited the government's ability to effectively deal with the pandemic. Though the economy's revival depends on herd immunity, Algeria seems to lag in vaccinating its citizens. This paper examines the current situation in Algeria, the challenges it faces, and the progress toward herd immunity, providing evidence for policymakers to promote a sustainable solution.

## IMPACTS OF COVID‐19 IN ALGERIA

**FIGURE 1:**
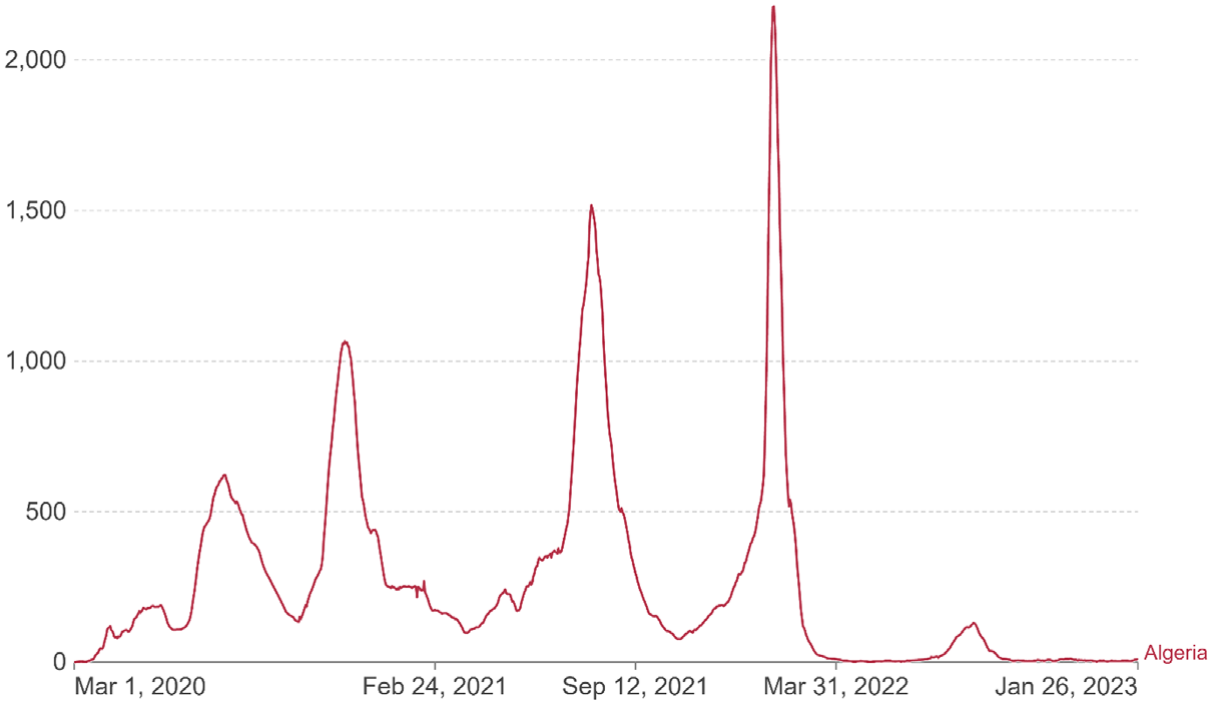
COVID‐19 cases in Algeria for the period of March 2020–January 2023.

Due to the ongoing COVID‐19 pandemic, Algeria is experiencing major challenges in its economy. To slow the spread of the virus, the government of Algeria began implementing measures on 13 March 2020. These measures included quarantining contacts of infected individuals, implementing contact tracing, closing schools and religious centers, and banning political protests and large gatherings. Additionally, wearing masks in public spaces became mandatory, and other measures, such as social distancing, closing workplaces except for essential workers, mandatory stay‐at‐home policies, quarantining new arrivals to the country, and travel bans, were also imposed [6]. However, despite these efforts, in January 2022, there was a spike in daily COVID‐19 cases, reaching an all‐time high of 2521 new cases per day. As a result, authorities had to take extra containment measures and impose a stricter lockdown. Since then, up to 150 daily new cases are detected. As of 24 January 2023, a total of 271, 354 confirmed cases and 6881 deaths had been reported by Our World in Data in Figure 1 [7].

Due to under‐testing, the actual number of COVID‐19 cases is likely to undercount the official numbers. This means that the number of reported cases may not adequately reflect the virus's true spread in the country. A study of spatiotemporal diffusion of COVID‐19 in Algeria found that the virus was suspected to have increased transmission in the northern parts of the country [8]. In a separate study, aimed to classify the risk of deaths and the spread of infections across various provinces in the country, several regions and areas were identified that required more attention and resources to control the spread of the virus [9]. Furthermore, another study highlighted the idea that the impact of the virus is not uniform across all cultural and ethnic groups [10]. Cultural practices, social norms, and economic conditions led to variations in the way the virus affected different cultural and ethnic groups, resulting in different rates of infection, morbidity, and mortality among them [10]. It is important to note that although these studies provide valuable insights, a more comprehensive study would be necessary to fully understand the dynamic spread of the virus across the country. These factors can make it challenging to achieve herd immunity in Algeria, as it is difficult to know the true number of people who have been infected. To achieve herd immunity, it is important to have accurate data on the number of cases, as well as vaccination efforts.

In April 2020, amidst the ongoing pandemic, oil stocks fell to an all‐time low due to low demand and a price war, which adversely affected countries like Algeria. Algeria relies heavily on oil exports and oil revenues, accounting for 98% of its foreign currency earnings and 50% of its tax revenues [12]. The weakness in economic activities also resulted in losses in several sectors, such as agriculture, leading to a decrease in tax contributions. For example, the Algerian airport corporation reported losses estimated at 3.1 billion Algerian dinars. To address the disruption of the supply chain, increase in prices, and unavailability of agricultural products, the Algerian government temporarily suspended the export of certain food and medical products in March 2020 [13]. This allowed them to have sufficient stock to cover local demand from March 2020 to the beginning of 2021. Additionally, the government opened several points of sale in different provinces where they sold certain agricultural products to citizens at economical prices to control the market price.

Conversely, a study reported that despite having a relatively well‐developed health system and spending a significant portion of gross domestic product on health care, Algeria struggled with preparedness for the COVID‐19 pandemic due to shortages of essential medical equipment and supplies, including oxygen, ventilators, intensive care unit beds, protective gear, and medications [2].

## STATE OF COVID‐19 VACCINATION

Algeria began its COVID19 vaccination campaign on 29 January 2021, with an initial shipment of 50,000 doses of the Sputnik V vaccine from Russia. In February 2021, the country received additional shipments of 50,000 doses of the Oxford‐AstraZeneca vaccine and 200,000 doses of the Sinopharm vaccine, donated by China. As of 4 September 2022, Algeria had received a total of 33,876,400 doses of COVID‐19 vaccines but had only administered 13,461,201, leaving over 60% of the vaccines unused. According to the Our World in Data project, as of 4 September 2022, only 30.2% of the population had received at least one dose of the vaccine, with 17.8% fully vaccinated [7].

**FIGURE 2:**
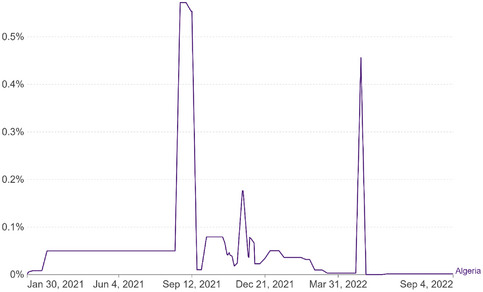
Daily share of the population receiving a COVID‐19 vaccine dose in Algeria.

The COVID‐19 Vaccines Global Access Program (COVAX) is a global initiative aimed at ensuring equitable access to COVID‐19 vaccines for all countries. As of September 2021, COVAX had supplied 15,926,400 AstraZeneca, Johnson & Johnson (J&J), and Sinovac vaccines to Algeria. The Algerian authorities also obtained 17,950,000 vaccine doses through bilateral arrangements and donations from AstraZeneca, Sinopharm, Sputnik V, and Sinovac [14]. Despite a peak in the daily vaccination rate of 256,927 in the first week of September 2021, the rate has since declined to 16,222 between 16 January and 8 February 2022, and an average of 14,207 since February 2022. However, only 18% of the population has received at least one dose of the vaccine, a low rate compared to the global average of 67.7%. Figure 2.

## CHALLENGES AND PROGRESS TOWARD HERD IMMUNITY

According to a report from World Health Organization (WHO) Africa, Algeria has fallen short of its vaccination goals, with only 34% of its population receiving at least one dose and just 17.8% fully vaccinated by 29 May 2022. This is significantly lower than the levels needed to achieve herd immunity [14]. The WHO had set a target for 40% of the population to be fully vaccinated by the end of 2021, and 70% by July 2022, but as of 4 September 2022, only 18.6% of Algeria's population had been fully vaccinated [7]. With a significant portion of the population remaining not vaccinated, with the reopening of the economy, the spread of the virus can be significantly increased, particularly among vulnerable populations [1].

Although safety concerns may be one factor contributing to vaccine hesitancy or regret, other factors may also be at play. These can include misinformation or lack of access to accurate information, mistrust of the government or pharmaceutical companies, or religious or cultural beliefs. It is also important to note that some studies conducted in Algeria have reported adverse events following vaccinations. These adverse events ranged from mild side effects such as pain or swelling at the injection site, to more serious side effects such as fever or allergic reactions [15, 16, 17]. Additionally, one of the studies reported that some individuals have regretted receiving the vaccines [17].

To overcome this set of disinformation, massive education and awareness campaigns should be launched to inform the public that, whereas adverse events to vaccines are possible, they are uncommon and the benefits of vaccination in preventing serious illness and death from COVID‐19 outweigh the risks. The WHO also confirms that vaccines have been rigorously tested and proven to be safe and effective in protecting against COVID‐19. Therefore, authorities must continue to monitor the situation and offer reliable vaccine information to the public, taking into account the social and cultural factors that may influence people's decisions to be vaccinated or not. By doing so, they can build trust and increase vaccination rates [18].

Another obstacle hindering Algeria's progress toward herd immunity is the government's lack of effective technology‐based strategies for vaccine distribution. These strategies can include online registration, Short Messaging Service reminders, and tracking vaccination status that can help to increase vaccine uptake and make it easier for individuals to access vaccines. The absence of these technology‐based strategies can make it difficult for the government to effectively reach out to all sections of its population [19].

## RECOMMENDATIONS

Although there has been some progress in reopening and recovering from the pandemic, the situation remains complex and uncertain with some countries disproportionately affected. To achieve herd immunity in Algeria, we suggest implementing the following strategies to increase the vaccination rate: improving vaccine distribution and access to remote communities through the use of technology such as drones. Prioritizing high‐risk groups, such as the elderly, immunocompromised individuals, front‐line health care workers, and essential service workers, can ensure that those most at risk of serious illness or death are protected first. Effective communication and engagement with the public are crucial for building trust in vaccines. The government should use digital tools, such as mobile apps and text message notifications, to inform people about vaccine availability and schedule appointments. Partnering with local pharmacies and retail businesses to establish vaccine clinics in convenient locations and investing in public education campaigns to increase awareness about the safety and effectiveness of vaccines can also help to increase vaccination uptake.

## CONCLUSION

Although the country has made some progress in vaccinating its population, there are significant challenges that need to be addressed, such as vaccine hesitancy and limited access to vaccines in certain areas. With only 13.5% of the population fully vaccinated, Algeria needs to develop policies and strategies to increase vaccination uptake to achieve herd immunity and restore normalcy.

## AUTHOR CONTRIBUTIONS

*Data curation; writing—original draft; writing—review and editing*: Chijioke Christopher Uhegwu and Oyinloye Emmanuel Abiodun. *Conceptualization; supervision; validation; writing—review and editing*: Usman Abubakar Haruna. *Supervision; validation; writing—review and editing*: Babaz Younes and Bakir Moussaouli. *Conceptualization; validation; writing—review and editing*: Goodness Ogeyi Odey.

## CONFLICT OF INTEREST STATEMENT

The authors declare no conflicts of interest.

## FUNDING INFORMATION

The authors declare that no funding was received for the development of this paper.

## ETHICS STATEMENT

There is no need for ethical approval.

## Data Availability

Data sharing not applicable to this article as no datasets were generated or analyzed during the current study.
